# A machine learning model for predicting the lymph node metastasis of early gastric cancer not meeting the endoscopic curability criteria

**DOI:** 10.1007/s10120-024-01511-8

**Published:** 2024-05-25

**Authors:** Minoru Kato, Yoshito Hayashi, Ryotaro Uema, Takashi Kanesaka, Shinjiro Yamaguchi, Akira Maekawa, Takuya Yamada, Masashi Yamamoto, Shinji Kitamura, Takuya Inoue, Shunsuke Yamamoto, Takashi Kizu, Risato Takeda, Hideharu Ogiyama, Katsumi Yamamoto, Kenji Aoi, Koji Nagaike, Yasutaka Sasai, Satoshi Egawa, Haruki Akamatsu, Hiroyuki Ogawa, Masato Komori, Nishihara Akihiro, Takeo Yoshihara, Yoshiki Tsujii, Tetsuo Takehara

**Affiliations:** 1 Department of Gastroenterology and Hepatology, Osaka University Graduate School of Medicine, Suita, Japan; 2 Department of Gastrointestinal Oncology, Osaka International Cancer Institute, Osaka, Japan; 3 Department of Gastroenterology, Kansai Rosai Hospital, Amagasaki, Japan; 4 Department of Internal Medicine, Osaka Police Hospital, Osaka, Japan; 5 Department of Gastroenterology, Osaka Rosai Hospital, Sakai, Japan; 6 Department of Gastroenterology, Toyonaka Municipal Hospital, Toyonaka, Japan; 7 Department of Gastroenterology, Sakai City Medical Center, Sakai, Japan; 8 Department of Gastroenterology, Osaka General Medical Center, Osaka, Japan; 9 Department of Gastroenterology, National Hospital Organization Osaka National Hospital, Osaka, Japan; 10 Department of Gastroenterology, Yao Municipal Hospital, Yao, Japan; 11 Department of Gastroenterology, Itami City Hospital, Itami, Japan; 12 Department of Gastroenterology, Ikeda Municipal Hospital, Ikeda, Japan; 13 Department of Gastroenterology, Japan Community Healthcare Organization Osaka Hospital, Osaka, Japan; 14 Department of Gastroenterology, Kaizuka City Hospital, Osaka, Japan; 15 Department of Gastroenterology, Suita Municipal Hospital, Suita, Japan; 16 Department of Gastroenterology, Otemae Hospital, Osaka, Japan; 17 Department of Gastroenterology, Kinki Central Hospital, Itami, Japan; 18 Department of Gastroenterology, Higashiosaka City Medical Center, Higashiosaka, Japan; 19 Department of Gastroenterology, Nishinomiya Municipal Central Hospital, Nishinomiya, Japan; 20 Department of Gastroenterology, Hyogo Prefectural Nishinomiya Hospital, Nishinomiya, Japan; 21 Department of Gastroenterology, Minoh City Hospital, Minoh, Japan

**Keywords:** Early gastric cancer, Endoscopic submucosal dissection, Lymph node metastasis, Machine learning, Artificial intelligence

## Abstract

**Background:**

We developed a machine learning (ML) model to predict the risk of lymph node metastasis (LNM) in patients with early gastric cancer (EGC) who did not meet the existing Japanese endoscopic curability criteria and compared its performance with that of the most common clinical risk scoring system, the eCura system.

**Methods:**

We used data from 4,042 consecutive patients with EGC from 21 institutions who underwent endoscopic submucosal dissection (ESD) and/or surgery between 2010 and 2021. All resected EGCs were histologically confirmed not to satisfy the current Japanese endoscopic curability criteria. Of all patients, 3,506 constituted the training cohort to develop the neural network-based ML model, and 536 constituted the validation cohort. The performance of our ML model, as measured by the area under the receiver operating characteristic curve (AUC), was compared with that of the eCura system in the validation cohort.

**Results:**

LNM rates were 14% (503/3,506) and 7% (39/536) in the training and validation cohorts, respectively. The ML model identified patients with LNM with an AUC of 0.83 (95% confidence interval, 0.76–0.89) in the validation cohort, while the eCura system identified patients with LNM with an AUC of 0.77 (95% confidence interval, 0.70–0.85) (*P* = 0.006, DeLong’s test).

**Conclusions:**

Our ML model performed better than the eCura system for predicting LNM risk in patients with EGC who did not meet the existing Japanese endoscopic curability criteria.

**Mini-abstract:**

We developed a neural network-based machine learning model that predicts the risk of lymph node metastasis in patients with early gastric cancer who did not meet the endoscopic curability criteria.

**Supplementary Information:**

The online version contains supplementary material available at 10.1007/s10120-024-01511-8.

## Introduction

Endoscopic submucosal dissection (ESD) is the standard treatment for early gastric cancer (EGC) in East Asia [1–5]. En bloc excision of cancer allows for a detailed histopathological evaluation, whereby treatment curability is determined. In the Japanese guidelines, when EGC resected by ESD does not fulfill the curability criteria, the resection is classified as endoscopic curability C (i.e., noncurative resection), which is further subclassified into endoscopic curability C-1 and C-2 [6]. Because the latter cases potentially have a risk of lymph node metastasis (LNM), additional surgery with lymphadenectomy is recommended. However, a recent meta-analysis reported that LNM was found in only 8.0% of patients with an endoscopic curability of C-2 [7]. As the risk of LNM varies among patients within the endoscopic curability C-2 group, subjecting all patients to additional surgery results in overtreatment. To minimize unnecessary additional surgeries, a precise prediction method for the LNM risk of EGCs categorized as endoscopic curability C-2 is needed.

For this purpose, we focused on machine learning (ML), which has been adopted to build accurate prediction models in various fields of medicine, including gastroenterology [8–12]. ML is a branch of artificial intelligence that uses algorithms to enable computers to learn automatically from data and determine the rules behind them. Once an ML algorithm is trained, it can predict unknown outcomes from new data with high accuracy. Currently, several scoring models stratify the risk of LNM in patients with EGC; however, all use conventional statistical analyses [13–16]. We hypothesized that ML models might perform better than existing models established using statistical analyses.

This study aimed to develop an ML-based risk prediction model for LNM in patients with EGC classified as endoscopic curability C-2 and compare its performance with that of the existing scoring model. Among the existing models, the “eCura system” is the most common risk-scoring model for LNM of EGC classified as endoscopic curability C-2 [14], and is currently recommended in the Japanese guidelines [6]. Hence, in this study, we chose this model for comparison.

## Methods

### Patients

This multicenter retrospective study was conducted at 21 institutions. The study was approved by the institutional review board of Osaka University (approval number: 22171, approval date: July 26, 2022) and the participating hospitals and was performed in accordance with the guidelines outlined in the Declaration of Helsinki.

We used the data of consecutive EGC patients who were treated with surgery, ESD with additional surgery, or ESD alone between 2010 and 2021 and were histologically confirmed as having endoscopic curability C-2. EGC was defined as an adenocarcinoma limited to the mucosa or submucosa, irrespective of LNM [17]. Exclusion criteria were as follows: special histological types of gastric cancer (e.g., neuroendocrine neoplasms, carcinoma with lymphoid stroma, adenocarcinoma of the fundic gland type [18, 19]), esophagogastric junction cancer, synchronous advanced cancer (in the stomach or other organs), synchronous EGC with endoscopic curability C-2, postoperative stomach, and missing data. Patients in the surgery group who had undergone preoperative chemotherapy were excluded. For the ESD-alone group, patients with follow-up periods < 3 years, not including patients who died of known causes within that time, or those who received adjuvant chemotherapy after ESD alone were excluded. Finally, cases with no lymphadenectomy in a surgical procedure (i.e., only local resection) were excluded even if the patients could be followed up for ≥ 3 years, because local resection of the stomach was described as an investigational treatment in Japanese gastric cancer treatment guidelines [6] and was not commonly performed. There was thus a possibility of taking an unusual course of events during the surveillance.

### Definition of endoscopic curability C-2

After endoscopic or surgical resection, histopathological evaluation was performed according to the Japanese classification system at each institution [17]. Specimens resected by ESD were sectioned at 2 mm intervals, whereas surgically resected specimens were sectioned at 5 mm intervals. Lymphovascular involvement was first examined by hematoxylin and eosin staining, and in cases with inconclusive findings, immunohistochemical staining was added.

Resected EGC was defined as under the curative state when it was resected in one piece, had no cancer-positive margins or lymphovascular involvement, and had one of the following conditions: (i) mucosal differentiated cancer with no ulceration; (ii) mucosal differentiated cancer with ulceration, ≤ 30 mm in diameter; (iii) undifferentiated, mucosal cancer without ulceration, ≤ 20 mm in diameter; or (iv) shallow (< 500 μm from the muscularis mucosae) submucosal differentiated cancer, ≤ 30 mm in diameter.

Otherwise, the resected EGC was considered to be in a state of endoscopic curability C (noncurative). If a positive horizontal margin was the only noncurative factor, it was categorized as endoscopic curability C-1. Other conditions were categorized as endoscopic curability C-2, and we only included patients with this histopathological character. The above-mentioned definition for the endoscopic curability C-2 was based on Japanese gastric cancer treatment guidelines [6].

### Data collection

The following data were collected: age, sex, tumor location, size, histological type, invasion depth, histopathological ulceration, lymphatic involvement, and vascular involvement. Histological types were classified as follows: (i) well-differentiated tubular adenocarcinoma (tub1); (ii) moderately differentiated tubular adenocarcinoma (tub2); (iii) papillary adenocarcinoma (pap); (iv) poorly differentiated adenocarcinoma (por); (v) signet-ring cell carcinoma (sig); and (vi) mucinous adenocarcinoma (muc). When more than one histological type was present in the tumor, the first two dominant histological types were collected in descending order (tub2 > tub1). Well-differentiated tubular adenocarcinoma (tub1), tub2, and pap were categorized as differentiated types, and por, sig, and muc were categorized as undifferentiated types. If the lesion had both types of cancer components, it was regarded as a mixed type. Invasion depth was classified into three categories: tumor limited to the mucosa (M), tumor invading the submucosa to a depth of < 500 μm from the muscularis mucosae (SM1), and tumor invading the submucosa to a depth ≥ 500 μm (SM2). Vertical margins were also investigated in patients who underwent ESD (with or without additional surgery). For the ESD-alone group, the development of metastatic recurrence in the lymph nodes and/or other organs during follow-up was also surveyed. Data were obtained from the medical records of each participating institution between August 2022 and December 2022.

### Definitions of outcome

The outcome selected to develop the ML model was LNM. For the surgery or ESD with additional surgery groups, it was defined as the presence of histologically identified metastases in the resected lymph nodes. For the ESD-alone group, it was defined as the development of metastatic recurrence in the lymph nodes and/or other organs diagnosed by computed tomography during follow-up. When patients in the ESD-alone group did not develop metastatic recurrence during a follow-up period of ≥ 3 years, LNM was considered negative. Patients with follow-up periods < 3 years were excluded from the ESD-alone group, except for those who died of known causes.

### Development of the ML model

We created two datasets: a training cohort used to build the ML model and a validation cohort used to compare the performance of the ML model with that of the eCura system. The former included all patient groups (surgery, ESD with additional surgery, or ESD alone), whereas the latter included only patients who underwent ESD (with or without additional surgery). The reasons for this were as follows: (i) the actual prediction target for our ML model and the eCura system were patients who underwent noncurative ESD, and (ii) in the eCura system, a positive vertical margin was set as a risk factor, which is assessable only in lesions resected by ESD. We randomly separated patients who underwent ESD (with or without additional surgery) into training and validation groups.

The ML model was constructed as a neural network with two hidden layers using Scikit-learn (https://scikit-learn.org), an ML library for Python. The training data were divided into four parts during the model training process, and parameter tuning was performed through fourfold cross-validation. We used the Adam optimizer for optimization. After parameter tuning, the first and second hidden layers comprised 6 and 18 nodes, respectively. The final inference model was an ensemble model (simple averaging) of the four models obtained through fourfold cross-validation. Hyperparameters of our ML model are listed in Supplementary Table S1.

For model development, we initially used age, sex, tumor location, lesion size, dominant histology, presence or absence of mixed-type histology, invasion depth, lymphatic involvement, vascular involvement, histopathological ulceration, vertical margin, and treatment method as input parameters. Through parameter tuning within the training dataset, the best predictions were achieved using the following seven factors: lesion size, dominant histology (tub2 or others), presence or absence of mixed-type histology, invasion depth (M, SM1, or SM2), lymphatic involvement (positive or negative), vascular involvement (positive or negative), and treatment method (surgery, or ESD with/without additional surgery). Most of our data were encoded as binary variable (i.e., 0 or 1) except for invasion depth and lesion size. For invasion depth, ordinal encoding was performed, such as 1 for SM2, 0.5 for SM1, and 0 for M. Lesion size was transformed to be in a range from 0 to 1 by dividing the raw data by 100.

### Statistical analysis

The Chi-squared and Fisher exact tests were used to compare categorical data, and the Kruskal–Wallis and Mann–Whitney *U* tests were used to compare continuous data. The area under the receiver operating characteristic curve (AUC) was used to measure the performance of the prediction models, and DeLong’s test was used to compare the AUCs. *P* values < 0.05 were considered statistically significant. Analyses were performed using JMP Pro version 16 (SAS Institute, Cary, NC, USA) or EZR version 1.61 (Saitama Medical Center, Jichi Medical University, Japan).

## Results

### Study cohort

**Fig. 1:**
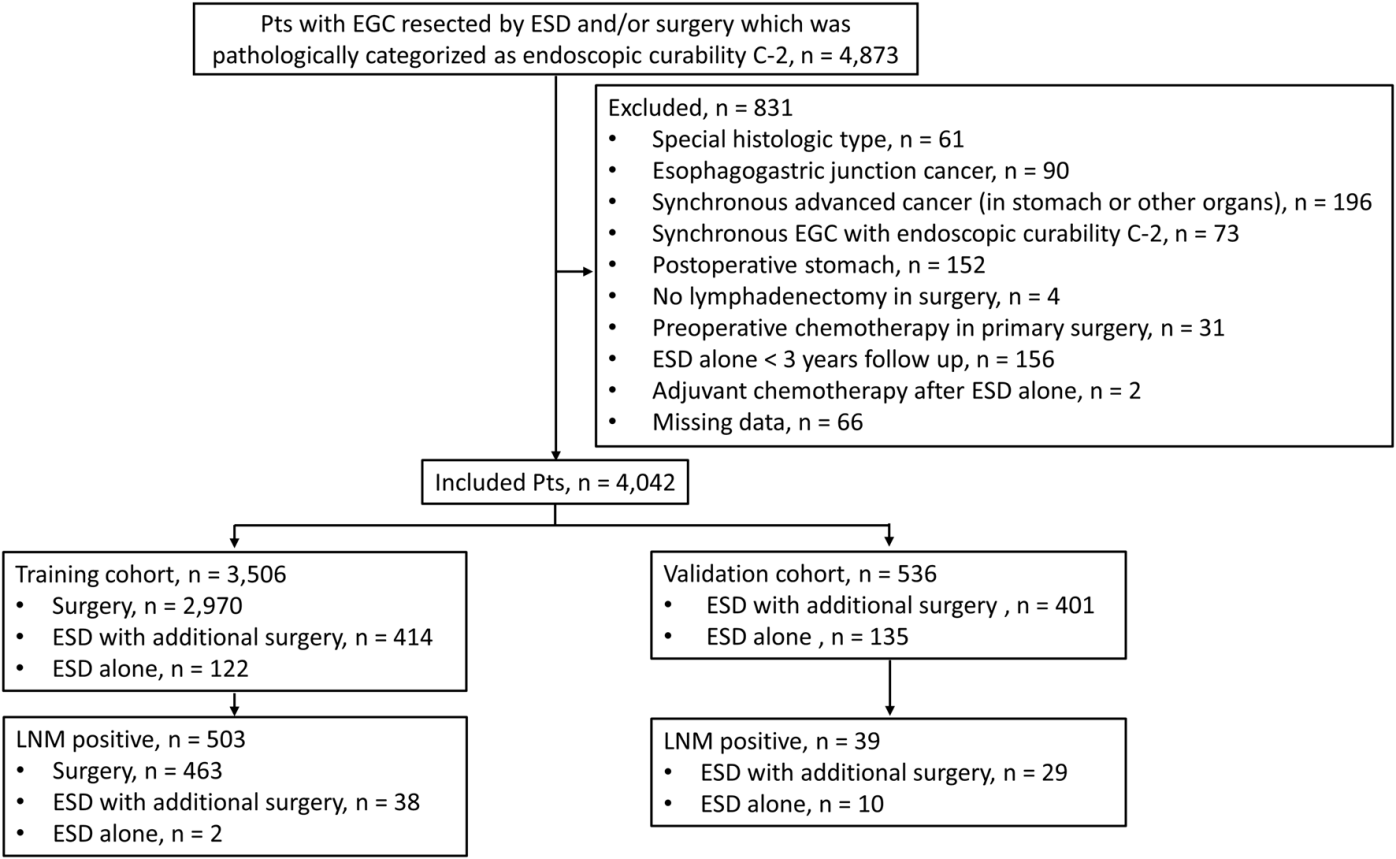
Patient selection flowchart. *Pts* patients; *ESD* endoscopic submucosal dissection; *EGC* early gastric cancer; *LNM* lymph node metastasis

Figure 1 shows the flowchart of patient selection. Among the 4,873 patients initially identified, 831 were excluded, and 4,042 were finally included: 3,506 patients in the training cohort and 536 patients in the validation cohort. In the training cohort, 2,970 patients (85%) underwent surgery, 414 (12%) underwent ESD with additional surgery, and 122 (3%) underwent ESD alone. In the validation cohort, 401 patients (75%) underwent ESD with additional surgery, and 135 (25%) underwent ESD alone. In the ESD-alone group, the median follow-up periods for the training and validation cohorts were 57 months (interquartile range [IQR] 41–73) and 55 months (IQR 41–74), respectively. Table 1 presents the characteristics of the training and validation cohorts. LNM was observed in 503 (14%) and 39 (7%) patients in the training and validation cohorts, respectively. The patient and lesion characteristics according to treatment are shown in Supplementary Table S2.

**Table 1:** Characteristics of the training and validation cohorts

	Training cohort	Validation cohort	*P* value
	*n* = 3506	*n* = 536	
Age, years	70 (26—94)	73 (37—90)	< 0.0001
Sex			< 0.0001
Male	2264 (65)	410 (76)	
Female	1242 (35)	126 (24)	
Treatment			
Surgery	2970 (85)	0 (0)	< 0.0001
ESD with additional surgery	414 (12)	401 (75)	
ESD alone	122 (3)	135 (25)	
Location			< 0.0001
Upper	582 (17)	130 (24)	
Middle	1689 (48)	226 (42)	
Lower	1235 (35)	180 (34)	
Size, mm	30 (2—185)	23 (5—103)	< 0.0001
Invasion depth			0.006
M	860 (25)	101 (19)	
SM1	418 (12)	80 (15)	
SM2	2228 (63)	355 (66)	
Histologic type			
Differentiated type			0.99
tub1 dominant	922 (26)	234 (44)	
tub2 dominant	514 (15)	132 (25)	
pap dominant	60 (2)	15 (3)	
Undifferentiated type			0.001
por dominant	778 (22)	29 (5)	
sig dominant	383 (11)	35 (7)	
muc dominant	11 (1)	0 (0)	
Mixed type			0.05
Differentiated type dominant	520 (15)	66 (12)	
Undifferentiated type dominant	318 (9)	25 (4)	
Lymphatic involvement			0.86
Positive	1183 (34)	183 (34)	
Negative	2323 (66)	353 (66)	
Vascular involvement			0.008
Positive	664 (19)	76 (14)	
Negative	2842 (81)	460 (86)	
Ulceration^a,b^			0.88
Positive	138 (28)	140 (28)	
Negative	352 (72)	365 (72)	
Vertical margin^a^			0.75
Positive	72 (13)	64 (12)	
Negative	432 (81)	438 (82)	
Unclear	32 (6)	34 (6)	
Lymph node metastasis			< 0.0001
Positive	503 (14)	39 (7)	
Negative	3003 (86)	497 (93)	

Data are expressed as the median (range) or number (%)

^a^ Shows only the results of the lesions resected by ESD with/without additional surgery

^b^ Data are unavailable for 46 and 31 lesions in the training and validation cohorts, respectively

*ESD* endoscopic submucosal dissection; *M* limited to the mucosa; *SM1* submucosal invasion < 500 μm; *SM2* submucosal invasion ≥ 500 μm; *tub1* well-differentiated tubular adenocarcinoma; *tub2* moderately differentiated tubular adenocarcinoma; *pap* papillary adenocarcinoma; *por* poorly differentiated adenocarcinoma; *sig* signet-ring cell carcinoma; *muc* mucinous adenocarcinoma

### Performance of the ML model

**Fig. 2:**
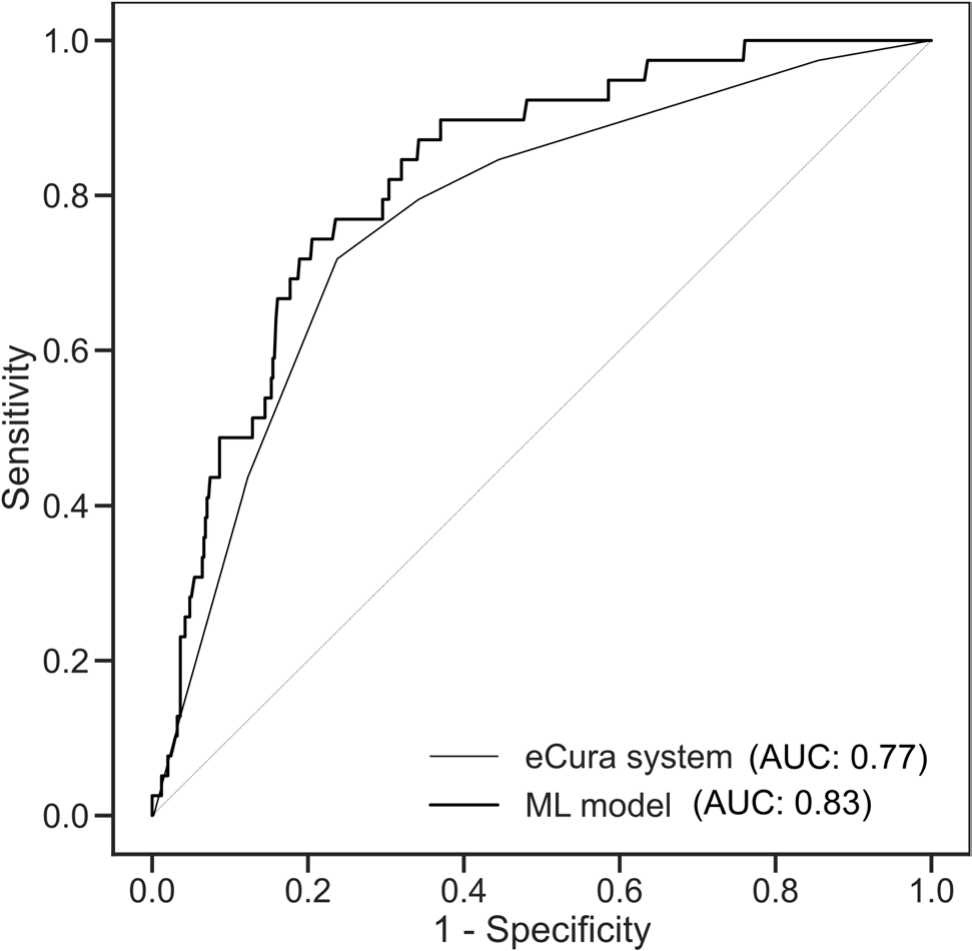
Receiver operating characteristic curves for the validation cohort (*n* = 536). *AUC* area under the curve

The ML model identified patients with LNM with an AUC of 0.83 [95% confidence interval (CI), 0.76–0.89] in the validation cohort, while the eCura system identified patients with LNM with an AUC of 0.77 (95% CI 0.70–0.85) (*P* = 0.006, DeLong’s test) (Fig. 2). At cutoff scores where the ML model and the eCura system identified patients with LNM with 100% sensitivity (i.e., a score of 0.02778 for the ML model and 0 for the eCura system), the specificity values were 24% (95% CI 20%–28%) for the ML model versus 0% (95% CI, 0.0%–1.1%) for the eCura system. This indicates that the ML model could reduce unnecessary surgery by up to 24% with a minimized risk of overlooking LNM, whereas no patients could avoid surgery with the eCura system.

**Fig. 3:**
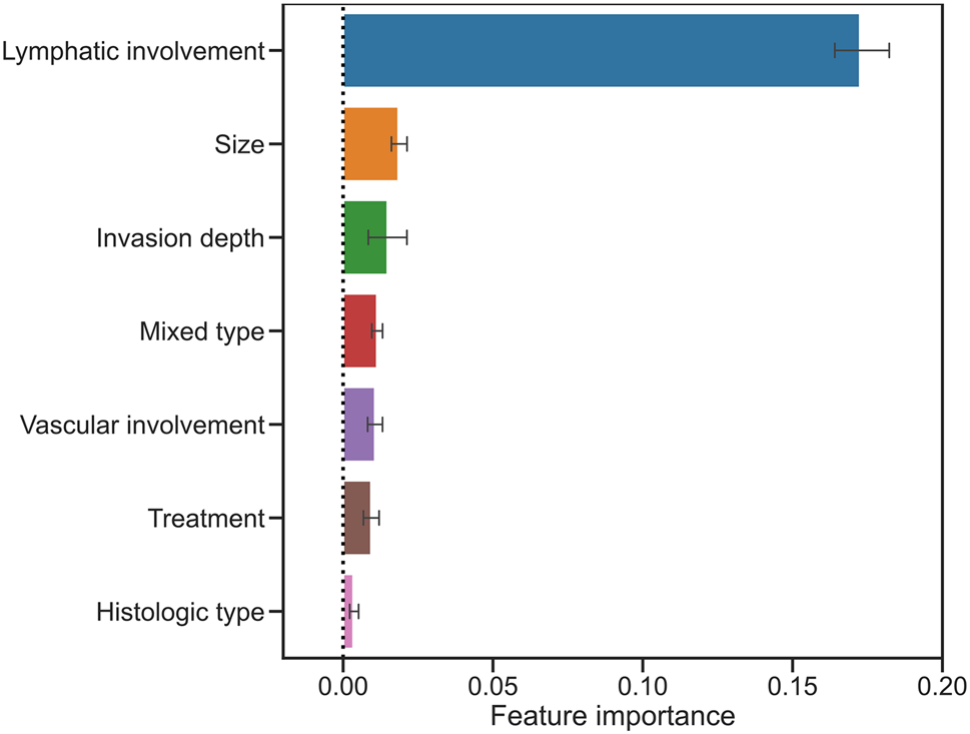
Permutation feature importance of the seven variables used to construct the machine learning model in the training cohort

The permutation feature importance of the seven variables used in the ML model was calculated for the training cohort (Fig. 3), and lymphatic involvement was found to be the most important factor for LNM.

### A web application of the ML model

We developed a web application to make our ML model freely available for clinicians (https://www.med.osaka-u.ac.jp/pub/gh/egc-lnm-prediction.html).

## Discussion

Our novel neural-network-based ML model derived from a large multi-institutional cohort identified the presence of LNM in patients with EGC categorized as endoscopic curability C-2 better than the most common risk-scoring model in Japan (i.e., the eCura system). Notably, the ML model performed better than the eCura system in choosing very low-risk patients who could be safely managed with only ESD. Our ML model has the potential to minimize unnecessary surgeries after gastric ESD.

Several researchers have developed ML models that predict the risk of LNM in patients with EGC [20–24]; however, these studies include many lesions satisfying the endoscopic curability criteria that have no risk of LNM. In contrast, we used only EGC data categorized as endoscopic curability C-2 (i.e., lesions at a high risk of LNM). Considering that prediction models are used for patients who are classified as endoscopic curability C-2 after gastric ESD, our ML model is more suitable and reliable than those previously reported.

Our study had following strengths. First, we directly compared the new ML model with the eCura system, the current most common risk-scoring model recommended presently in the Japanese gastric cancer treatment guidelines [6]. The eCura system was developed based on data from patients who underwent surgery after noncurative ESD [14]. Hence, we excluded patients who underwent surgery as the first treatment from the validation cohort to allow the eCura system to demonstrate its true performance. In this fair situation, our ML model showed a significantly higher AUC than that of the eCura system (0.83 versus 0.77, *P* = 0.006). The predictive ability of the eCura system shown in this study (AUC of 0.77) was almost the same as the original results shown by the developers (Hatta W, et al.) (AUC of 0.74) [14], which guarantees the credibility of our results. Second, we included information on the presence of mixed-type histology in the ML model because it is a potential predictor of LNM in EGC [13, 25–27]. In fact, analysis of feature importance showed mixed-type histology as the fourth-most important factor in our model (Fig. 3). Since the eCura system does not evaluate information about mixed-type histology, we believe that this difference conferred better results with our model.

One of the problems with the eCura system was that the number of undifferentiated-type EGCs, which are often treated by primary surgery, was small in the development cohort (14%, 150/1101 cases). Thus, Hatta et al*.* reported that the risk of undifferentiated-type histology may be underestimated in the eCura system [28]. As a measure for this problem, we decided to include primary surgical cases in the training cohort. As a result, we could increase the number of patients with undifferentiated-type EGC (40%, 1490/3506 cases). It might be ideal to increase the number of patients with undifferentiated-type EGC using only ESD cases. However, due to the limited number of ESD cases available, we decided to use primary surgical cases as an alternative.

Although a positive vertical margin is regarded as a risk factor for LNM in the eCura system, we did not include this factor in our ML model because it did not improve the predictive power (data not shown). This might be because we used many surgery cases in the training cohort in which the vertical margin was not evaluable.

We classified the histologic types of EGC into two groups, tub2 or others, for our final ML model. Other classifications, such as differentiated versus undifferentiated, did not show better performance. One reason for this could be that tub2 was the most frequent histologic type among LNM-positive EGCs (41%, 204/503) in the training cohort in this study.

When our ML model is used in clinical settings, the worst scenario is to overlook LNM because it may eventually cause metastatic recurrence. Once this occurs, salvage surgery is almost impossible and can be fatal [29]. Therefore, we chose the cutoff score of the ML model by setting the sensitivity to 100% in the validation cohort. At 100% sensitivity, the ML model had a specificity of 24%, while the eCura system had a specificity of 0%. This means that among the 497 patients who did not have LNM in the validation cohort, the ML model could help 120 patients (24%) avoid unnecessary surgery, whereas none (0%) could avoid unnecessary surgery with the eCura system. Thus, our ML model performed better than the eCura system in correctly identifying patients who did not require surgery after ESD. Characteristics of those 120 patients who could have avoided additional surgery by our ML model (i.e., true negatives in our ML model) is shown in Supplementary Table S3. The scores of the eCura system in those patients were 0 point for 49 patients (41%), 1 point for 68 patients (57%), and 2 points for 3 patients (2%). No patients neither had eCura scores of ≥ 3 points, nor lymphatic involvement (Table S3).

Our study had several limitations. First, the sectioning interval of the resected specimen differed between the ESD (2 mm) and surgery (5 mm) groups. Histopathological evaluation of surgically resected specimens carries the risk of underestimating the invasion depth and overlooking lymphatic and/or vascular involvement because of the wide sectioning interval. As a result, the effect of each risk factor on LNM may differ between the ESD and surgery groups. To minimize this problem, we included “treatment method” as an input parameter in the process of training, making the ML model learn those differences between the ESD and surgery groups. Second, the immunohistochemical staining for assessing lymphatic and vascular involvement was not performed for all the cases. This might also have caused the underestimation of the lymphatic and/or vascular involvement. Third, vertical cancer margin was not evaluable in surgically resected specimens. Fourth, the rate of patients with LNM-positive EGC in the validation cohort (7%) was smaller than that of the training cohort (14%). Fifth, regarding the ESD-alone group, whether a minimum of 3 years of follow-up was sufficient remains controversial. However, considering that metastatic recurrence often appears within 3 years after EGC resection [30], we believe our follow-up period was acceptable. Sixth, we did not collect information on the extent of lymph node dissection or the number of resected lymph nodes in patients who underwent surgery, which may differ according to preoperative staging. Some patients might have undergone insufficient lymph node dissection, causing an underestimation of LNM; however, our large cohort might have reduced this bias.

In conclusion, we developed an ML model that performed better than the eCura system in predicting the risk of LNM in patients with EGC who did not meet the Japanese endoscopic curability criteria. This precision model is potentially useful for minimizing unnecessary surgeries after gastric ESD. A prospective study is required to further validate our ML model.

### Supplementary Information

Below is the link to the electronic supplementary material.

Supplementary file1 (PDF 2964 KB)

## References

[1] Kato M, Nishida T, Tsutsui S, Komori M, Michida T, Yamamoto K, et al. Endoscopic submucosal dissection as a treatment for gastric noninvasive neoplasia: a multicenter study by Osaka University ESD Study Group. J Gastroenterol. 2011;46:325–31. 21107615 10.1007/s00535-010-0350-1

[2] Akasaka T, Nishida T, Tsutsui S, Michida T, Yamada T, Ogiyama H, et al. Short-term outcomes of endoscopic submucosal dissection (ESD) for early gastric neoplasm: multicenter survey by Osaka University ESD study group. Dig Endosc. 2011;23:73–7. 21198921 10.1111/j.1443-1661.2010.01062.x

[3] Shichijo S, Uedo N, Kanesaka T, Ohta T, Nakagawa K, Shimamoto Y, et al. Long-term outcomes after endoscopic submucosal dissection for differentiated-type early gastric cancer that fulfilled expanded indication criteria: a prospective cohort study. J Gastroenterol Hepatol. 2021;36:664–70. 32663347 10.1111/jgh.15182 PMC7983953

[4] Chung IK, Lee JH, Lee SH, Kim SJ, Cho JY, Cho WY, et al. Therapeutic outcomes in 1000 cases of endoscopic submucosal dissection for early gastric neoplasms: Korean ESD Study Group multicenter study. Gastrointest Endosc. 2009;69:1228–35. 19249769 10.1016/j.gie.2008.09.027

[5] Song WC, Qiao XL, Gao XZ. A comparison of endoscopic submucosal dissection (ESD) and radical surgery for early gastric cancer: a retrospective study. World J Surg Oncol. 2015;13:309. 26537433 10.1186/s12957-015-0724-1 PMC4634741

[6] Japanese Gastric Cancer Association. Japanese gastric cancer treatment guidelines 2021 (6th edition). Gastric Cancer. 2023;26:1–25. 36342574 10.1007/s10120-022-01331-8 PMC9813208

[7] Hatta W, Gotoda T, Kanno T, Yuan Y, Koike T, Moayyedi P, et al. Prevalence and risk factors for lymph node metastasis after noncurative endoscopic resection for early gastric cancer: a systematic review and meta-analysis. J Gastroenterol. 2020;55:742–53. 32277297 10.1007/s00535-020-01685-9

[8] Ichimasa K, Kudo SE, Mori Y, Misawa M, Matsudaira S, Kouyama Y, et al. Artificial intelligence may help in predicting the need for additional surgery after endoscopic resection of T1 colorectal cancer. Endoscopy. 2018;50:230–40. 29272905 10.1055/s-0043-122385

[9] Shung DL, Au B, Taylor RA, Tay JK, Laursen SB, Stanley AJ, et al. Validation of a machine learning model that outperforms clinical risk scoring systems for upper gastrointestinal bleeding. Gastroenterology. 2020;158:160–7. 31562847 10.1053/j.gastro.2019.09.009 PMC7004228

[10] Kudo SE, Ichimasa K, Villard B, Mori Y, Misawa M, Saito S, et al. Artificial intelligence system to determine risk of T1 colorectal cancer metastasis risk to lymph node. Gastroenterology. 2021;160:1075-1084.e2. 32979355 10.1053/j.gastro.2020.09.027

[11] Arai J, Aoki T, Sato M, Niikura R, Suzuki N, Ishibashi R, et al. Machine learning-based personalized prediction of gastric cancer incidence using the endoscopic and histologic findings at the initial endoscopy. Gastrointest Endosc. 2022;95:864–72. 34998795 10.1016/j.gie.2021.12.033

[12] Ichimasa K, Nakahara K, Kudo SE, Misawa M, Bretthauer M, Shimada S, et al. Novel “resect and analysis” approach for T2 colorectal cancer with use of artificial intelligence. Gastrointest Endosc. 2022;96:665-672.e1. 35500659 10.1016/j.gie.2022.04.1305

[13] Sekiguchi M, Oda I, Taniguchi H, Suzuki H, Morita S, Fukagawa T, et al. Risk stratification and predictive risk-scoring model for lymph node metastasis in early gastric cancer. J Gastroenterol. 2016;51:961–70. 26884381 10.1007/s00535-016-1180-6

[14] Hatta W, Gotoda T, Oyama T, Kawata N, Takahashi A, Yoshifuku Y, et al. A scoring system to stratify curability after endoscopic submucosal dissection for early gastric cancer: “eCura system.” Am J Gastroenterol. 2017;112:874–81. 28397873 10.1038/ajg.2017.95

[15] Kim SM, Min BH, Ahn JH, Jung SH, An JY, Choi MG, et al. Nomogram to predict lymph node metastasis in patients with early gastric cancer: a useful clinical tool to reduce gastrectomy after endoscopic resection. Endoscopy. 2020;52:435–43. 32162286 10.1055/a-1117-3059

[16] Cai F, Dong Y, Wang P, Zhang L, Yang Y, Liu Y, et al. Risk assessment of lymph node metastasis in early gastric cancer: establishment and validation of a seven-point scoring model. Surgery. 2022;171:1273–80. 34865863 10.1016/j.surg.2021.10.049

[17] Japanese Gastric Cancer Association. Japanese classification of gastric carcinoma: 3rd English edition. Gastric Cancer English ed. 2011;14:101–12. 10.1007/s10120-011-0041-5 21573743

[18] Ueyama H, Yao T, Nakashima Y, Hirakawa K, Oshiro Y, Hirahashi M, et al. Gastric adenocarcinoma of fundic gland type (chief cell predominant type): proposal for a new entity of gastric adenocarcinoma. Am J Surg Pathol. 2010;34:609–19. 20410811 10.1097/PAS.0b013e3181d94d53

[19] WHO Classification of Tumours Editorial Board. WHO classification of tumours 5th ed Vol 1, Digestive system tumours. Lyon: IARC; 2019.

[20] Lee HD, Nam KH, Shin CM, Lee HS, Chang YH, Yoon H, et al. Development and validation of models to predict lymph node metastasis in early gastric cancer using logistic regression and gradient boosting machine methods. Cancer Res Treat. 2023;55:1240–9. 36960625 10.4143/crt.2022.1330 PMC10582533

[21] Yang T, Martinez-Useros J, Liu J, Alarcón I, Li C, Li W, et al. A retrospective analysis based on multiple machine learning models to predict lymph node metastasis in early gastric cancer. Front Oncol. 2022;12:1023110. 36530978 10.3389/fonc.2022.1023110 PMC9751349

[22] Na JE, Lee YC, Kim TJ, Lee H, Won HH, Min YW, et al. Machine learning model to stratify the risk of lymph node metastasis for early gastric cancer: a single-center cohort study. Cancers. 2022;14:1121. 35267429 10.3390/cancers14051121 PMC8909118

[23] Zhu H, Wang G, Zheng J, Zhu H, Huang J, Luo E, et al. Preoperative prediction for lymph node metastasis in early gastric cancer by interpretable machine learning models: a multicenter study. Surgery. 2022;171:1543–51. 35131110 10.1016/j.surg.2021.12.015

[24] Tian H, Ning Z, Zong Z, Liu J, Hu C, Ying H, et al. Application of machine learning algorithms to predict lymph node metastasis in early gastric cancer. Front Med (Lausanne). 2022;8: 759013. 35118083 10.3389/fmed.2021.759013 PMC8806156

[25] Hanaoka N, Tanabe S, Mikami T, Okayasu I, Saigenji K. Mixed-histologic-type submucosal invasive gastric cancer as a risk factor for lymph node metastasis: feasibility of endoscopic submucosal dissection. Endoscopy. 2009;41:427–32. 19418397 10.1055/s-0029-1214495

[26] Takizawa K, Ono H, Kakushima N, Tanaka M, Hasuike N, Matsubayashi H, et al. Risk of lymph node metastases from intramucosal gastric cancer in relation to histological types: how to manage the mixed histological type for endoscopic submucosal dissection. Gastric Cancer. 2013;16:531–6. 23192620 10.1007/s10120-012-0220-z

[27] Lee JH, Choi IJ, Han HS, Kim YW, Ryu KW, Yoon HM, et al. Risk of lymph node metastasis in differentiated type mucosal early gastric cancer mixed with minor undifferentiated type histology. Ann Surg Oncol. 2015;22:1813–9. 25344305 10.1245/s10434-014-4167-7

[28] Hatta W, Gotoda T, Oyama T, Kawata N, Takahashi A, Yoshifuku Y, et al. Is the eCura system useful for selecting patients who require radical surgery after noncurative endoscopic submucosal dissection for early gastric cancer? A comparative study Gastric Cancer. 2018;21:481–9. 28983696 10.1007/s10120-017-0769-7

[29] Takizawa K, Hatta W, Gotoda T, Kawata N, Nakagawa M, Takahashi A, et al. Recurrence patterns and outcomes of salvage surgery in cases of non-curative endoscopic submucosal dissection without additional radical surgery for early gastric cancer. Digestion. 2019;99:52–8. 30554228 10.1159/000494413

[30] Saka M, Katai H, Fukagawa T, Nijjar R, Sano T. Recurrence in early gastric cancer with lymph node metastasis. Gastric Cancer. 2008;11:214–8. 19132483 10.1007/s10120-008-0485-4

