# Supplementary material for: A machine learning model for predicting the lymph node metastasis of early gastric cancer not meeting the endoscopic curability criteria
Source: Gastric Cancer. 2024 May 25;27(5):1069–77. doi: 10.1007/s10120-024-01511-8 (PMC11335823; doi:10.1007/s10120-024-01511-8)
Supplement: Supplementary file 1 — Supplementary file1 (PDF 2964 KB) [file 10120_2024_1511_MOESM1_ESM.pdf]

Gastric Cancer

A machine learning model for predicting the lymph node metastasis of early gastric cancer not meeting the endoscopic curability criteria

--Manuscript Draft--

**Manuscript Number:**	GCAN-D-23-00774R2
**Full Title:**	A machine learning model for predicting the lymph node metastasis of early gastric cancer not meeting the endoscopic curability criteria
**Article Type:**	Original article
**Manuscript Classifications:**	1.003: Endoscopy; 2.010: EMR/ESD; 2.012: Prognostic factors
**Keywords:**	early gastric cancer; endoscopic submucosal dissection; lymph node metastasis; Machine Learning; Artificial intelligence
**Corresponding Author:**	Tetsuo Takehara, M.D., Ph.D<br>Department of Gastroenterology and Hepatology, Osaka University Graduate School of Medicine.<br>Suita, Osaka JAPAN
**Corresponding Author Secondary Information:**	
**Corresponding Author's Institution:**	Department of Gastroenterology and Hepatology, Osaka University Graduate School of Medicine.
**Corresponding Author's Secondary Institution:**	
**First Author:**	Minoru Kato
**First Author Secondary Information:**	
**Order of Authors:**	Minoru Kato
	Yoshito Hayashi
	Ryotaro Uema
	Takashi Kanesaka
	Shinjiro Yamaguchi
	Akira Maekawa
	Takuya Yamada
	Masashi Yamamoto
	Shinji Kitamura
	Takuya Inoue
	Shunsuke Yamamoto
	Takashi Kizu
	Risato Takeda
	Hideharu Ogiyama
	Katsumi Yamamoto
	Kenji Aoi
	Koji Nagaike
	Yasutaka Sasai
	Satoshi Egawa
	Haruki Akamatsu

Powered by Editorial Manager® and ProduXion Manager® from Aries Systems Corporation

	Hiroyuki Ogawa
	Masato Komori
	Nishihara Akihiro
	Takeo Yoshihara
	Yoshiki Tsujii
	Tetsuo Takehara, M.D., Ph.D
**Order of Authors Secondary Information:**	
**Funding Information:**	
**Abstract:**	Background: We developed a machine learning (ML) model to predict the risk of lymph node metastasis (LNM) in patients with early gastric cancer (EGC) who did not meet the existing Japanese endoscopic curability criteria and compared its performance with that of the most common clinical risk scoring system, the eCura system.<br>Methods: We used data from 4,042 consecutive patients with EGC from 21 institutions who underwent endoscopic submucosal dissection (ESD) and/or surgery between 2010 and 2021. All resected EGCs were histologically confirmed not to satisfy the current Japanese endoscopic curability criteria. Of all patients, 3,506 constituted the training cohort to develop the neural network–based ML model, and 536 constituted the validation cohort. The performance of our ML model, as measured by the area under the receiver operating characteristic curve (AUC), was compared with that of the eCura system in the validation cohort.<br>Results: LNM rates were 14% (503/3,506) and 7% (39/536) in the training and validation cohorts, respectively. The ML model identified patients with LNM with an AUC of 0.83 (95% confidence interval, 0.76-0.89) in the validation cohort, while the eCura system identified patients with LNM with an AUC of 0.77 (95% confidence interval, 0.70-0.85) (P = 0.006, DeLong's test).<br>Conclusions: Our ML model performed better than the eCura system for predicting LNM risk in patients with EGC who did not meet the existing Japanese endoscopic curability criteria.
**Additional Information:**	
**Question**	**Response**
Is the work reported on in your paper a clinical study?	No
Is the work reported on in your paper a prospective study?	No
Have you already obtained approval for your work from the IRB (Institutional Review Board)?	Yes
Do you agree to submit the original protocol upon request from the editorial committee?	Yes

Powered by Editorial Manager® and ProduXion Manager® from Aries Systems Corporation

Responses to the reviewer's comments.

Reviewer: 1

(1) The issue previously raised as Major (1)

Using only ESD specimens as training data is not a matter of selection bias. Rather, it should be selected as the optimal training dataset. The reason for this is as mentioned in the previous comment, to develop a more precise and effective ML system, uniformity is needed between the quality of training and validation dataset. Given ESD group is set for validation cohort, the only ESD group should be chosen for training dataset.

Regarding the article reported by Hatta W et al., choosing only ESD specimens was not a mistake, but the study design was appropriate. The problem is the small sample size due to the rarity of the disease and the difference in the criteria of additional resection between differentiated and undifferentiated gastric cancer. Then, the statement "To solve this problem, we included surgery cases in the training cohort" is not an optimal solution because the study design, which was ideally set up in "Hatta study", has collapsed.

However, it is understandable that inclusion of surgery-cases was necessary due to the number of ESD cases available. My previous comment that surgical specimens should ideally be excluded from the test data did not mean that the current study should be re-conducted in that way. Nor am I suggesting that surgical specimens should be included in the validation cohort. The author's decision to exclude them from the validation was the correct one. However, the limitation of this study is that surgery-cases could not be excluded from the training-data. It is important to correctly understand what is ideal, to recognize that the current method was chosen in consideration of realistic numbers, and to fairly reflect it as a limitation.

Therefore, their response ("however, we believe that the advantage of reducing selection bias is more important for the risk assessment of LNM.") is not an appropriate description. The correct interpretation is that the training data should also be ESD samples as well as validation data, but surgical samples were added to the training data in order to prioritize the sample size of undifferentiated gastric cancers, and this should be mentioned. I recommend that you read the previous comment carefully and reflect the above.

Reply

Thank you for your insightful comment. We understand your concerns very well. We

reflected your comment in the Discussion section as follows.

DISCUSSION, page 18 line 10-18.

"One of the problems with the eCura system was that the number of undifferentiated-type EGCs, which are often treated by primary surgery, was small in the development cohort (14%, 150/1101 cases). Thus, Hatta et al. reported that the risk of undifferentiated-type histology may be underestimated in the eCura system [28]. As a measure for this problem, we decided to include primary surgical cases in the training cohort. As a result, we could increase the number of patients with undifferentiated-type EGC (40%, 1490/3506 cases). It might be ideal to increase the number of patients with undifferentiated-type EGC using only ESD cases. However, due to the limited number of ESD cases available, we decided to use primary surgical cases as an alternative."

(2) The issue previously raised as Major (5)

Although Table 1 is well edited, it is insufficient in Table 2. Was multiple comparison test used for? If so, it should be mentioned, including; which pairwise comparison showed significance and what method was applied to the analysis, such as Bonferroni Correction.

Reply

Thank you for your important comment. To indicate the differences between the three groups, we used Chi-square test and Kruskal-Wallis test. We did not perform multiple pairwise comparisons, and therefore the Bonferroni Correction was not performed. We concern that multiple pairwise comparisons may complicate the TableS2 and will not provide meaningful information to the readers.

(3) The issue previously raised as Major (6)

It is good editing to add the limitation to the document, however, the last sentence "however, it was sufficient for statistical analyses." is inappropriate. Considering appropriate sample size for external validation, 39 events are insufficient for statistical analysis. Please delete this.

Reply

Thank you for your important comment. We deleted the sentence, “however, it was sufficient for statistical analyses.”, from the Discussion section.

April 11th 2024
Prof. Yasuhiro Kodera
Editor in Chief
Gastric Cancer

Dear Prof. Yasuhiro Kodera

We would like to thank you for allowing us to resubmit our manuscript (GCAN-D-23-00774R1) entitled “A machine learning model for predicting the lymph node metastasis of early gastric cancer not meeting the endoscopic curability criteria”. The comments of the reviewers have been very helpful in allowing us to revise our manuscript. We have revised according to the reviewers’ comments, and provided a point-by-point reply to each comment. As you can see, we agreed with most of the reviewers’ comments and modified the original manuscript accordingly.
Thank you for your consideration of the revised version. Please consider this manuscript for the publication in Gastric Cancer.
Thank you.

Sincerely yours,

Tetsuo Takehara, MD, PhD
Department of Gastroenterology and Hepatology,
Osaka University Graduate School of Medicine,
2-2 Yamadaoka Suita, Osaka 565-0871, Japan.
Tel: +81-6-6879-3621; Fax: +81-6-6879-3629; E-mail: takehara@gh.med.osaka-u.ac.jp

Manuscript

Click here to view linked References

Click here to access/download;Manuscript;Maindocument_Minoru

Original Article

A machine learning model for predicting the lymph node metastasis of early gastric cancer not meeting the endoscopic curability criteria

Minoru Kato[1,2], Yoshito Hayashi[1], Ryotaro Uema[1], Takashi Kanesaka[2], Shinjiro Yamaguchi[3], Akira Maekawa[4], Takuya Yamada[5], Masashi Yamamoto[6], Shinji Kitamura[7], Takuya Inoue[8], Shunsuke Yamamoto[9], Takashi Kizu[10], Risato Takeda[11], Hideharu Ogiyama[12], Katsumi Yamamoto[13], Kenji Aoi[14], Koji Nagaike[15], Yasutaka Sasai[16], Satoshi Egawa[17], Haruki Akamatsu[18], Hiroyuki Ogawa[19], Masato Komori[20], Nishihara Akihiro[21], Takeo Yoshihara[1], Yoshiki Tsujii[1], Tetsuo Takehara[1].

[1] Department of Gastroenterology and Hepatology, Osaka University Graduate School of Medicine, Suita, Japan

[2] Department of Gastrointestinal Oncology, Osaka International Cancer Institute, Osaka, Japan

[3] Department of Gastroenterology, Kansai Rosai Hospital, Amagasaki, Japan

[4] Department of Internal Medicine, Osaka Police Hospital, Osaka, Japan

[5] Department of Gastroenterology, Osaka Rosai Hospital, Sakai, Japan

[6] Department of Gastroenterology, Toyonaka Municipal Hospital, Toyonaka, Japan

[7] Department of Gastroenterology, Sakai City Medical Center, Sakai, Japan

[8] Department of Gastroenterology, Osaka General Medical Center, Osaka, Japan.

[9] Department of Gastroenterology, National Hospital Organization Osaka National Hospital, Osaka, Japan

[10] Department of Gastroenterology, Yao Municipal Hospital, Yao, Japan

[11] Department of Gastroenterology, Itami City Hospital, Itami, Japan

[12] Department of Gastroenterology, Ikeda Municipal Hospital, Ikeda, Japan

[13] Department of Gastroenterology, Japan Community Healthcare Organization Osaka Hospital, Osaka, Japan

[14] Department of Gastroenterology, Kaizuka City Hospital, Osaka, Japan

[15] Department of Gastroenterology, Suita Municipal Hospital, Suita, Japan

[16] Department of Gastroenterology, Otemae Hospital, Osaka, Japan

[17] Department of Gastroenterology, Kinki Central Hospital, Itami, Japan

[18] Department of Gastroenterology, Higashiosaka City Medical Center, Higashiosaka, Japan

[19] Department of Gastroenterology, Nishinomiya Municipal Central Hospital,

Nishinomiya, Japan

[20] Department of Gastroenterology, Hyogo Prefectural Nishinomiya Hospital, Nishinomiya, Japan

[21] Department of Gastroenterology, Minoh City Hospital, Minoh, Japan

Correspondence: Tetsuo Takehara, MD, PhD, Department of Gastroenterology and Hepatology, Osaka University Graduate School of Medicine, 2-2 Yamadaoka Suita, Osaka 565-0871, Japan

Tel: +81-6-6879-3621; Fax: +81-6-6879-3629; E-mail: takehara@gh.med.osaka-u.ac.jp

Short running head: ML model for predicting LNM in EGC

Guarantor of the article: Tetsuo Takehara

Specific author contributions:

Conception and design: Minoru Kato, Yoshito Hayashi, Ryotaro Uema, Yoshiki Tsujii.

Data collection: Minoru Kato, Takashi Kanesaka, Shinjiro Yamaguchi, Akira Maekawa, Takuya Yamada, Masashi Yamamoto, Shinji Kitamura, Takuya Inoue, Shunsuke

Yamamoto, Takashi Kizu, Risato Takeda, Hideharu Ogiyama, Katsumi Yamamoto, Kenji Aoi, Koji Nagaike, Yasutaka Sasai, Satoshi Egawa, Haruki Akamatsu, Hiroyuki Ogawa, Masato Komori and Nishihara Akihiro.

Development of machine learning model: Ryotaro Uema.

Data analysis and interpretation: Minoru Kato, Ryotaro Uema.

Drafting of the article: Minoru Kato, Ryotaro Uema.

Critical revision: Yoshito Hayashi, Tetsuo Takehara.

Final approval of the article: All authors.

Financial support: None to report.

Conflict of Interest: The authors declare that they have no conflict of interest.

Word count: 3,044 words

ABSTRACT

Background: We developed a machine learning (ML) model to predict the risk of lymph node metastasis (LNM) in patients with early gastric cancer (EGC) who did not meet the existing Japanese endoscopic curability criteria and compared its performance with that of the most common clinical risk scoring system, the eCura system.

Methods: We used data from 4,042 consecutive patients with EGC from 21 institutions who underwent endoscopic submucosal dissection (ESD) and/or surgery between 2010 and 2021. All resected EGCs were histologically confirmed not to satisfy the current Japanese endoscopic curability criteria. Of all patients, 3,506 constituted the training cohort to develop the neural network–based ML model, and 536 constituted the validation cohort. The performance of our ML model, as measured by the area under the receiver operating characteristic curve (AUC), was compared with that of the eCura system in the validation cohort.

Results: LNM rates were 14% (503/3,506) and 7% (39/536) in the training and validation cohorts, respectively. The ML model identified patients with LNM with an AUC of 0.83 (95% confidence interval, 0.76-0.89) in the validation cohort, while the eCura system identified patients with LNM with an AUC of 0.77 (95% confidence interval, 0.70-0.85) (P = 0.006, DeLong's test).

Conclusions: Our ML model performed better than the eCura system for predicting LNM risk in patients with EGC who did not meet the existing Japanese endoscopic curability criteria.

Keywords: early gastric cancer, endoscopic submucosal dissection, lymph node metastasis, machine learning, artificial intelligence

Mini-abstract: We developed a neural network-based machine learning model that predicts the risk of lymph node metastasis in patients with early gastric cancer who did not meet the endoscopic curability criteria.

INTRODUCTION

Endoscopic submucosal dissection (ESD) is the standard treatment for early gastric cancer (EGC) in East Asia [1-5]. En bloc excision of cancer allows for a detailed histopathological evaluation, whereby treatment curability is determined. In the Japanese guidelines, when EGC resected by ESD does not fulfil the curability criteria, the resection is classified as endoscopic curability C (i.e., noncurative resection), which is further subclassified into endoscopic curability C-1 and C-2 [6]. Because the latter cases potentially have a risk of lymph node metastasis (LNM), additional surgery with lymphadenectomy is recommended. However, a recent meta-analysis reported that LNM was found in only 8.0% of patients with an endoscopic curability of C-2 [7]. As the risk of LNM varies among patients within the endoscopic curability C-2 group, subjecting all patients to additional surgery results in overtreatment. To minimize unnecessary additional surgeries, a precise prediction method for the LNM risk of EGCs categorized as endoscopic curability C-2 is needed.

For this purpose, we focused on machine learning (ML), which has been adopted to build accurate prediction models in various fields of medicine, including gastroenterology [8-12]. ML is a branch of artificial intelligence that uses algorithms to enable computers to learn automatically from data and determine the rules behind them.

Once an ML algorithm is trained, it can predict unknown outcomes from new data with high accuracy. Currently, several scoring models stratify the risk of LNM in patients with EGC; however, all use conventional statistical analyses [13-16]. We hypothesised that ML models might perform better than existing models established using statistical analyses.

This study aimed to develop an ML-based risk prediction model for LNM in patients with EGC classified as endoscopic curability C-2 and compare its performance with that of the existing scoring model. Among the existing models, the "eCura system" is the most common risk-scoring model for LNM of EGC classified as endoscopic curability C-2 [14], and is currently recommended in the Japanese guidelines [6]. Hence, in this study, we chose this model for comparison.

METHODS

Patients

This multicenter retrospective study was conducted at 21 institutions. The study was approved by the institutional review board of Osaka University (approval number: 22171, approval date: July 26, 2022) and the participating hospitals and was performed in accordance with the guidelines outlined in the Declaration of Helsinki.

We used the data of consecutive EGC patients who were treated with surgery, ESD with additional surgery, or ESD alone between 2010 and 2021 and were histologically confirmed as having endoscopic curability C-2. EGC was defined as an adenocarcinoma limited to the mucosa or submucosa, irrespective of LNM [17]. Exclusion criteria were as follows: special histological types of gastric cancer (e.g., neuroendocrine neoplasms, carcinoma with lymphoid stroma, adenocarcinoma of the fundic gland type [18, 19]), esophagogastric junction cancer, synchronous advanced cancer (in the stomach or other organs), synchronous EGC with endoscopic curability C-2, postoperative stomach, and missing data. Patients in the surgery group who had undergone preoperative chemotherapy were excluded. For the ESD-alone group, patients with follow-up periods < 3 years, not including patients who died of known causes within that time, or those who received adjuvant chemotherapy after ESD alone were excluded. Finally, cases with no lymphadenectomy in a surgical procedure (i.e., only local resection) were excluded even if the patients could be followed up for ≥ 3 years, because local resection of the stomach was described as an investigational treatment in Japanese gastric cancer treatment guidelines [6], and was not commonly performed. There was thus a possibility of taking an unusual course of events during the surveillance.

Definition of endoscopic curability C-2

After endoscopic or surgical resection, histopathological evaluation was performed according to the Japanese classification system at each institution [17]. Specimens resected by ESD were sectioned at 2 mm intervals, whereas surgically resected specimens were sectioned at 5 mm intervals. Lymphovascular involvement was first examined by hematoxylin and eosin staining, and in cases with inconclusive findings, immunohistochemical staining was added.

Resected EGC was defined as under the curative state when it was resected in one piece, had no cancer-positive margins or lymphovascular involvement, and had one of the following conditions: (i) mucosal differentiated cancer with no ulceration; (ii) mucosal differentiated cancer with ulceration, ≤ 30 mm in diameter; (iii) undifferentiated, mucosal cancer without ulceration, ≤ 20 mm in diameter; or (iv) shallow (< 500 μm from the muscularis mucosae) submucosal differentiated cancer, ≤ 30 mm in diameter.

Otherwise, the resected EGC was considered to be in a state of endoscopic curability C (noncurative). If a positive horizontal margin was the only noncurative factor, it was categorized as endoscopic curability C-1. Other conditions were categorized as endoscopic curability C-2, and we only included patients with this histopathological

character. The abovementioned definition for the endoscopic curability C-2 was based on Japanese gastric cancer treatment guidelines [6].

Data collection

The following data were collected: age, sex, tumor location, size, histological type, invasion depth, histopathological ulceration, lymphatic involvement, and vascular involvement. Histological types were classified as follows: (i) well-differentiated tubular adenocarcinoma (tub1); (ii) moderately differentiated tubular adenocarcinoma (tub2); (iii) papillary adenocarcinoma (pap); (iv) poorly differentiated adenocarcinoma (por); (v) signet-ring cell carcinoma (sig), and (vi) mucinous adenocarcinoma (muc). When more than one histological type was present in the tumor, the first two dominant histological types were collected in descending order (tub2 > tub1). Well-differentiated tubular adenocarcinoma (tub1), tub2, and pap were categorized as differentiated types, and por, sig, and muc were categorized as undifferentiated types. If the lesion had both types of cancer components, it was regarded as a mixed type. Invasion depth was classified into three categories: tumor limited to the mucosa (M), tumor invading the submucosa to a depth of < 500 μm from the muscularis mucosae (SM1), and tumor invading the submucosa to a depth ≥ 500 μm (SM2). Vertical margins were also investigated in patients

who underwent ESD (with or without additional surgery). For the ESD-alone group, the development of metastatic recurrence in the lymph nodes and/or other organs during follow-up was also surveyed. Data were obtained from the medical records of each participating institution between August 2022 and December 2022.

Definitions of outcome

The outcome selected to develop the ML model was LNM. For the surgery or ESD with additional surgery groups, it was defined as the presence of histologically identified metastases in the resected lymph nodes. For the ESD alone group, it was defined as the development of metastatic recurrence in the lymph nodes and/or other organs diagnosed by computed tomography during follow-up. When patients in the ESD alone group did not develop metastatic recurrence during a follow-up period of ≥ 3 years, LNM was considered negative. Patients with follow-up periods < 3 years were excluded from the ESD alone group, except for those who died of known causes.

Development of the ML model

We created two datasets: a training cohort used to build the ML model and a validation cohort used to compare the performance of the ML model with that of the eCura system.

The former included all patient groups (surgery, ESD with additional surgery, or ESD alone), whereas the latter included only patients who underwent ESD (with or without additional surgery). The reasons for this were as follows: (i) the actual prediction target for our ML model and the eCura system were patients who underwent noncurative ESD, and (ii) in the eCura system, a positive vertical margin was set as a risk factor, which is assessable only in lesions resected by ESD. We randomly separated patients who underwent ESD (with or without additional surgery) into training and validation groups.

The ML model was constructed as a neural network with two hidden layers using Scikit-learn (https://scikit-learn.org), an ML library for Python. The training data were divided into four parts during the model training process, and parameter tuning was performed through 4-fold cross-validation. We used the Adam optimizer for optimization. After parameter tuning, the first and second hidden layers comprised 6 and 18 nodes, respectively. The final inference model was an ensemble model (simple averaging) of the four models obtained through 4-fold cross-validation. Hyperparameters of our ML model are listed in Supplementary Table S1.

For model development, we initially used age, sex, tumor location, lesion size, dominant histology, presence or absence of mixed-type histology, invasion depth, lymphatic involvement, vascular involvement, histopathological ulceration, vertical

margin, and treatment method as input parameters. Through parameter tuning within the training dataset, the best predictions were achieved using the following seven factors: lesion size, dominant histology (tub2 or others), presence or absence of mixed-type histology, invasion depth (M, SM1, or SM2), lymphatic involvement (positive or negative), vascular involvement (positive or negative), and treatment method (surgery, or ESD with/without additional surgery). Most of our data were encoded as binary variable (i.e., 0 or 1) except for invasion depth and lesion size. For invasion depth, ordinal encoding was performed, such as 1 for SM2, 0.5 for SM1, and 0 for M. Lesion size was transformed to be in a range from 0 to 1 by dividing the raw data by 100.

Statistical analysis

The chi-squared and Fisher exact tests were used to compare categorical data, and the Kruskal-Wallis and Mann-Whitney U tests were used to compare continuous data. The area under the receiver operating characteristic curve (AUC) was used to measure the performance of the prediction models, and DeLong's test was used to compare the AUCs. P values < 0.05 were considered statistically significant. Analyses were performed using JMP Pro version 16 (SAS Institute, Cary, NC, USA) or EZR version 1.61 (Saitama Medical Center, Jichi Medical University, Japan).

RESULTS

Study cohort

Figure 1 shows the flow chart of patient selection. Among the 4,873 patients initially identified, 831 were excluded, and 4,042 were finally included: 3,506 patients in the training cohort and 536 patients in the validation cohort. In the training cohort, 2,970 patients (85%) underwent surgery, 414 (12%) underwent ESD with additional surgery, and 122 (3%) underwent ESD alone. In the validation cohort, 401 patients (75%) underwent ESD with additional surgery, and 135 (25%) underwent ESD alone. In the ESD alone group, the median follow-up periods for the training and validation cohorts were 57 months (interquartile range [IQR] 41-73) and 55 months (IQR 41-74), respectively. Table 1 presents the characteristics of the training and validation cohorts. LNM was observed in 503 (14%) and 39 (7%) patients in the training and validation cohorts, respectively. The patient and lesion characteristics according to treatment are shown in Supplementary Table S2.

Performance of the ML model

The ML model identified patients with LNM with an AUC of 0.83 [95% confidence

interval (CI), 0.76-0.89] in the validation cohort, while the eCura system identified patients with LNM with an AUC of 0.77 (95% CI, 0.70-0.85) (P = 0.006, DeLong's test) (Fig. 2). At cut-off scores where the ML model and the eCura system identified patients with LNM with 100% sensitivity (i.e., a score of 0.02778 for the ML model and 0 for the eCura system), the specificity values were 24% (95% CI, 20%-28%) for the ML model versus 0% (95% CI, 0.0%-1.1%) for the eCura system. This indicates that the ML model could reduce unnecessary surgery by up to 24% with a minimized risk of overlooking LNM, whereas no patients could avoid surgery with the eCura system.

The permutation feature importance of the seven variables used in the ML model was calculated for the training cohort (Fig. 3), and lymphatic involvement was found to be the most important factor for LNM.

A web application of the ML model

We developed a web application to make our ML model freely available for clinicians (https://www.med.osaka-u.ac.jp/pub/gh/egc-lnm-prediction.html).

DISCUSSION

Our novel neural-network-based ML model derived from a large multi-institutional

cohort identified the presence of LNM in patients with EGC categorized as endoscopic curability C-2 better than the most common risk-scoring model in Japan (i.e., the eCura system). Notably, the ML model performed better than the eCura system in choosing very low-risk patients who could be safely managed with only ESD. Our ML model has the potential to minimize unnecessary surgeries after gastric ESD.

Several researchers have developed ML models that predict the risk of LNM in patients with EGC [20-24]; however, these studies include many lesions satisfying the endoscopic curability criteria that have no risk of LNM. In contrast, we used only EGC data categorized as endoscopic curability C-2 (i.e., lesions at a high risk of LNM). Considering that prediction models are used for patients who are classified as endoscopic curability C-2 after gastric ESD, our ML model is more suitable and reliable than those previously reported.

Our study had following strengths. First, we directly compared the new ML model with the eCura system, the current most common risk-scoring model recommended presently in the Japanese gastric cancer treatment guidelines [6]. The eCura system was developed based on data from patients who underwent surgery after noncurative ESD [14]. Hence, we excluded patients who underwent surgery as the first treatment from the validation cohort to allow the eCura system to demonstrate its true performance. In this

fair situation, our ML model showed a significantly higher AUC than that of the eCura system (0.83 versus 0.77, $P = 0.006$). The predictive ability of the eCura system shown in this study (AUC of 0.77) was almost the same as the original results shown by the developers (Hatta W, et al.) (AUC of 0.74) [14], which guarantees the credibility of our results. Second, we included information on the presence of mixed-type histology in the ML model because it is a potential predictor of LNM in EGC [13, 25-27]. In fact, analysis of feature importance showed mixed-type histology as the fourth most important factor in our model (Fig. 3). Since the eCura system does not evaluate information about mixed-type histology, we believe that this difference conferred better results with our model.

One of the problems with the eCura system was that the number of undifferentiated-type EGCs, which are often treated by primary surgery, was small in the development cohort (14%, 150/1101 cases). Thus, Hatta et al. reported that the risk of undifferentiated-type histology may be underestimated in the eCura system [28]. As a measure for this problem, we decided to include primary surgical cases in the training cohort. As a result, we could increase the number of patients with undifferentiated-type EGC (40%, 1490/3506 cases). It might be ideal to increase the number of patients with undifferentiated-type EGC using only ESD cases. However, due to the limited number of ESD cases available, we decided to use primary surgical cases as an alternative.

Although a positive vertical margin is regarded as a risk factor for LNM in the eCura system, we did not include this factor in our ML model because it did not improve the predictive power (data not shown). This might be because we used many surgery cases in the training cohort in which the vertical margin was not evaluable.

We classified the histologic types of EGC into two groups, tub2 or others, for our final ML model. Other classifications, such as differentiated versus undifferentiated, did not show better performance. One reason for this could be that tub2 was the most frequent histologic type among LNM-positive EGCs (41%, 204/503) in the training cohort in this study.

When our ML model is used in clinical settings, the worst scenario is to overlook LNM because it may eventually cause metastatic recurrence. Once this occurs, salvage surgery is almost impossible and can be fatal [29]. Therefore, we chose the cut-off score of the ML model by setting the sensitivity to 100% in the validation cohort. At 100% sensitivity, the ML model had a specificity of 24%, while the eCura system had a specificity of 0%. This means that among the 497 patients who did not have LNM in the validation cohort, the ML model could help 120 patients (24%) avoid unnecessary surgery, whereas none (0%) could avoid unnecessary surgery with the eCura system. Thus, our ML model performed better than the eCura system in correctly identifying patients who

did not require surgery after ESD. Characteristics of those 120 patients who could have avoided additional surgery by our ML model (i.e., true negatives in our ML model) is shown in Supplementary Table S3. The scores of the eCura system in those patients were 0 point for 49 patients (41%), 1 point for 68 patients (57%), and 2 points for 3 patients (2%). No patients neither had eCura scores of ≥ 3 points, nor lymphatic involvement (Table S3).

Our study had several limitations. First, the sectioning interval of the resected specimen differed between the ESD (2 mm) and surgery (5 mm) groups. Histopathological evaluation of surgically resected specimens carries the risk of underestimating the invasion depth and overlooking lymphatic and/or vascular involvement because of the wide sectioning interval. As a result, the effect of each risk factor on LNM may differ between ESD and surgery groups. To minimize this problem, we included "treatment method" as an input parameter in the process of training, making ML model learn those difference between ESD and surgery groups. Second, the immunohistochemical staining for assessing lymphatic and vascular involvement was not performed for all the cases. This might also have caused the underestimation of the lymphatic and/or vascular involvement. Third, vertical cancer margin was not evaluable in surgically resected specimens. Fourth, the rate of patients with LNM-positive EGC in

the validation cohort (7%) was smaller than that of the training cohort (14%). Fifth, regarding the ESD-alone group, whether a minimum of 3 years of follow-up was sufficient remains controversial. However, considering that metastatic recurrence often appears within three years after EGC resection [30], we believe our follow-up period was acceptable. Sixth, we did not collect information on the extent of lymph node dissection or the number of resected lymph nodes in patients who underwent surgery, which may differ according to preoperative staging. Some patients might have undergone insufficient lymph node dissection, causing an underestimation of LNM; however, our large cohort might have reduced this bias.

In conclusion, we developed an ML model that performed better than the eCura system in predicting the risk of LNM in patients with EGC who did not meet the Japanese endoscopic curability criteria. This precision model is potentially useful for minimizing unnecessary surgeries after gastric ESD. A prospective study is required to further validate our ML model.

ACKNOWLEDGEMENTS

The authors wish to express their gratitude to Drs. Ayaka Tajiri, Hiromu Fukuda, Eiji Kimura, Kentaro Nakagawa, Hirotsugu Saiki, (Osaka University), Shunsuke Yoshii, Satoki Shichijo, Sachiko Yamamoto, Yoji Takeuchi, Koji Higashino, Noriya Uedo, Ryu Ishihara, Tomoki Michida (Osaka International Cancer Institute), Takanori Inoue, Takashi Ohta, Hideki Hagiwara (Kansai Rosai Hospital), Akihiko Sakatani, Hideki Iijima (Osaka Police Hospital), Yukihiro Kusumoto, Naoki Hiramatsu (Osaka Rosai Hospital), Dai Nakamatsu, Tsutomu Nishida (Toyonaka Municipal Hospital), Narihiro Shibukawa (Sakai City Medical Center), Takayuki Yakushijin (Osaka General Medical Center), Ryotaro Sakamori (National Hospital Organization Osaka National Hospital), Mitsuru Sakakibara, Fukui Hiroyuki (Yao Municipal Hospital), Shusaku Tsutsui (Itami City Hospital), Yoshitaka Yamaguchi, Akira Tategami (Ikeda Municipal Hospital), Akira Kaneko (Japan Community Health Care Organization Osaka Hospital), Naruyasu Kakita (Kaizuka City Hospital), Kengo Nagai, Yuichi Yoshida, Masafumi Naito (Suita Municipal Hospital), Yoshinori Doi (Otemae Hospital), Akinori Shimayoshi (Kinki Central Hospital), Ichizo Kobayashi, Masahiko Tsujii (Higashiosaka City Medical Center), Sadaharu Iio (Hyogo Prefectural Nishinomiya Hospital), Masanori Nakahara (Minoh City

Hospital) for significant assistance with preparation for the study. We would like to thank Editage (www.editage.com) for English language editing.

References

1. Kato M, Nishida T, Tsutsui S, Komori M, Michida T, Yamamoto K, et al. Endoscopic submucosal dissection as a treatment for gastric noninvasive neoplasia: a multicenter study by Osaka University ESD Study Group. J Gastroenterol. 2011;46:325–31.
2. Akasaka T, Nishida T, Tsutsui S, Michida T, Yamada T, Ogiyama H, et al. Short-term outcomes of endoscopic submucosal dissection (ESD) for early gastric neoplasm: multicenter survey by Osaka University ESD study group. Dig Endosc. 2011;23:73–7.
3. Shichijo S, Uedo N, Kanesaka T, Ohta T, Nakagawa K, Shimamoto Y, et al. Long-term outcomes after endoscopic submucosal dissection for differentiated-type early gastric cancer that fulfilled expanded indication criteria: A prospective cohort study. J Gastroenterol Hepatol. 2021;36:664–70.
4. Chung IK, Lee JH, Lee SH, Kim SJ, Cho JY, Cho WY, et al. Therapeutic outcomes in 1000 cases of endoscopic submucosal dissection for early gastric neoplasms: Korean ESD Study Group multicenter study. Gastrointest Endosc. 2009;69:1228–35.
5. Song WC, Qiao XL, Gao XZ. A comparison of endoscopic submucosal dissection (ESD) and radical surgery for early gastric cancer: a retrospective study. World J Surg Oncol. 2015;13:309.

6. Japanese Gastric Cancer Association. Japanese gastric cancer treatment guidelines 2021 (6th edition). Gastric Cancer. 2023;26:1–25.

7. Hatta W, Gotoda T, Kanno T, Yuan Y, Koike T, Moayyedi P, et al. Prevalence and risk factors for lymph node metastasis after noncurative endoscopic resection for early gastric cancer: a systematic review and meta-analysis. J Gastroenterol. 2020;55:742–53.

8. Ichimasa K, Kudo SE, Mori Y, Misawa M, Matsudaira S, Kouyama Y, et al. Artificial intelligence may help in predicting the need for additional surgery after endoscopic resection of T1 colorectal cancer. Endoscopy. 2018;50:230–40.

9. Shung DL, Au B, Taylor RA, Tay JK, Laursen SB, Stanley AJ, et al. Validation of a machine learning model that outperforms clinical risk scoring systems for upper gastrointestinal bleeding. Gastroenterology. 2020;158:160–7.

10. Kudo SE, Ichimasa K, Villard B, Mori Y, Misawa M, Saito S, et al. Artificial intelligence system to determine risk of T1 colorectal cancer metastasis risk to lymph node. Gastroenterology. 2021;160:1075–1084.e2.

11. Arai J, Aoki T, Sato M, Niikura R, Suzuki N, Ishibashi R, et al. Machine learning-based personalized prediction of gastric cancer incidence using the endoscopic and histologic findings at the initial endoscopy. Gastrointest Endosc. 2022;95:864–72.

12. Ichimasa K, Nakahara K, Kudo SE, Misawa M, Bretthauer M, Shimada S, et al. Novel “resect and analysis” approach for T2 colorectal cancer with use of artificial intelligence. Gastrointest Endosc. 2022;96:665–672.e1.
13. Sekiguchi M, Oda I, Taniguchi H, Suzuki H, Morita S, Fukagawa T, et al. Risk stratification and predictive risk-scoring model for lymph node metastasis in early gastric cancer. J Gastroenterol. 2016;51:961–70.
14. Hatta W, Gotoda T, Oyama T, Kawata N, Takahashi A, Yoshifuku Y, et al. A scoring system to stratify curability after endoscopic submucosal dissection for early gastric cancer: “eCura system”. Am J Gastroenterol. 2017;112:874–81.
15. Kim SM, Min BH, Ahn JH, Jung SH, An JY, Choi MG, et al. Nomogram to predict lymph node metastasis in patients with early gastric cancer: a useful clinical tool to reduce gastrectomy after endoscopic resection. Endoscopy. 2020;52:435–43.
16. Cai F, Dong Y, Wang P, Zhang L, Yang Y, Liu Y, et al. Risk assessment of lymph node metastasis in early gastric cancer: establishment and validation of a seven-point scoring model. Surgery. 2022;171:1273–80.
17. Japanese Gastric Cancer Association. Japanese classification of gastric carcinoma: 3rd English edition. Gastric Cancer. English ed. 2011;14:101–12.
18. Ueyama H, Yao T, Nakashima Y, Hirakawa K, Oshiro Y, Hirahashi M, et al. Gastric

adenocarcinoma of fundic gland type (chief cell predominant type): proposal for a new entity of gastric adenocarcinoma. Am J Surg Pathol. 2010;34:609–19.

19. WHO Classification of Tumours Editorial Board. WHO classification of tumours. 5th ed. Vol. 1, Digestive system tumours. Lyon: IARC; 2019.

20. Lee HD, Nam KH, Shin CM , Lee HS, Chang YH, Yoon H, et al. Development and validation of models to predict lymph node metastasis in early gastric cancer using logistic regression and gradient boosting machine methods. Cancer Res Treat. 2023;55:1240–49.

21. Yang T, Martinez-Useros J, Liu J, Alarcón I, Li C, Li W, et al. A retrospective analysis based on multiple machine learning models to predict lymph node metastasis in early gastric cancer. Front Oncol. 2022;12:1023110.

22. Na JE, Lee YC, Kim TJ, Lee H, Won HH, Min YW, et al. Machine learning model to stratify the risk of lymph node metastasis for early gastric cancer: A single-center cohort study. Cancers. 2022;14:1121.

23. Zhu H, Wang G, Zheng J, Zhu H, Huang J, Luo E, et al. Preoperative prediction for lymph node metastasis in early gastric cancer by interpretable machine learning models: A multicenter study. Surgery. 2022;171:1543–51.

24. Tian H, Ning Z, Zong Z, Liu J, Hu C, Ying H, et al. Application of machine learning

algorithms to predict lymph node metastasis in early gastric cancer. Front Med (Lausanne). 2022;8:759013.

25. Hanaoka N, Tanabe S, Mikami T, Okayasu I, Saigenji K. Mixed-histologic-type submucosal invasive gastric cancer as a risk factor for lymph node metastasis: feasibility of endoscopic submucosal dissection. Endoscopy. 2009;41:427–32.

26. Takizawa K, Ono H, Kakushima N, Tanaka M, Hasuike N, Matsubayashi H, et al. Risk of lymph node metastases from intramucosal gastric cancer in relation to histological types: how to manage the mixed histological type for endoscopic submucosal dissection. Gastric Cancer. 2013;16:531–6.

27. Lee JH, Choi IJ, Han HS, Kim YW, Ryu KW, Yoon HM, et al. Risk of lymph node metastasis in differentiated type mucosal early gastric cancer mixed with minor undifferentiated type histology. Ann Surg Oncol. 2015;22:1813–9.

28. Hatta W, Gotoda T, Oyama T, Kawata N, Takahashi A, Yoshifuku Y, et al. Is the eCura system useful for selecting patients who require radical surgery after noncurative endoscopic submucosal dissection for early gastric cancer? A comparative study. Gastric Cancer. 2018;21:481–9.

29. Takizawa K, Hatta W, Gotoda T, Kawata N, Nakagawa M, Takahashi A, et al. Recurrence patterns and outcomes of salvage surgery in cases of non-curative

endoscopic submucosal dissection without additional radical surgery for early gastric cancer. Digestion. 2019;99:52–8.

30. Saka M, Katai H, Fukagawa T, Nijjar R, Sano T. Recurrence in early gastric cancer with lymph node metastasis. Gastric Cancer. 2008;11:214–8.

Table 1. Characteristics of the training and validation cohorts

	Training cohort	Validation cohort	*P* value
	n = 3506	n = 536	
Age, years	70 (26 - 94)	73 (37 - 90)	< 0.0001
Sex			< 0.0001
Male	2264 (65)	410 (76)	
Female	1242 (35)	126 (24)	
Treatment			
Surgery	2970 (85)	0 (0)	< 0.0001
ESD with additional surgery	414 (12)	401 (75)	
ESD alone	122 (3)	135 (25)	
Location			< 0.0001
Upper	582 (17)	130 (24)	
Middle	1689 (48)	226 (42)	
Lower	1235 (35)	180 (34)	
Size, mm	30 (2 - 185)	23 (5 - 103)	< 0.0001
Invasion depth			0.006
M	860 (25)	101 (19)	
SM1	418 (12)	80 (15)	
SM2	2228 (63)	355 (66)	
Histologic type			
Differentiated type			0.99
tub1 dominant	922 (26)	234 (44)	
tub2 dominant	514 (15)	132 (25)	
pap dominant	60 (2)	15 (3)	
Undifferentiated type			0.001
por dominant	778 (22)	29 (5)	
sig dominant	383 (11)	35 (7)	
muc dominant	11 (1)	0 (0)	
Mixed type			0.05
Differentiated type dominant	520 (15)	66 (12)	
Undifferentiated type dominant	318 (9)	25 (4)	
Lymphatic involvement			0.86
Positive	1183 (34)	183 (34)	
Negative	2323 (66)	353 (66)	

Vascular involvement			0.008
Positive	664 (19)	76 (14)	
Negative	2842 (81)	460 (86)	
Ulceration [a, b]			0.88
Positive	138 (28)	140 (28)	
Negative	352 (72)	365 (72)	
Vertical margin [a]			0.75
Positive	72 (13)	64 (12)	
Negative	432 (81)	438 (82)	
Unclear	32 (6)	34 (6)	
Lymph node metastasis			< 0.0001
Positive	503 (14)	39 (7)	
Negative	3003 (86)	497 (93)	

Data are expressed as the median (range) or number (%).

[a] Shows only the results of the lesions resected by ESD with/without additional surgery.

[b] Data are unavailable for 46 and 31 lesions in the training and validation cohorts, respectively.

ESD, endoscopic submucosal dissection; M, limited to the mucosa; SM1, submucosal invasion < 500 µm; SM2, submucosal invasion≥ 500µm; tub1, well-differentiated tubular adenocarcinoma; tub2, moderately differentiated tubular adenocarcinoma; pap, papillary adenocarcinoma; por, poorly differentiated adenocarcinoma; sig, signet-ring cell carcinoma; muc, mucinous adenocarcinoma

Figure Legends

Fig. 1 Patient selection flowchart. Pts, patients; ESD, endoscopic submucosal dissection; EGC, early gastric cancer; LNM, lymph node metastasis.

Fig. 2 Receiver operating characteristic curves for the validation cohort (n = 536). AUC, Area under the curve.

Fig. 3 Permutation feature importance of the seven variables used to construct the machine learning model in the training cohort.

Table S1. Hyperparameters of ML model

Hyperparameter	Value
Classifier model	MLPClassifier
Hidden_layer_sizes	(6, 18)
Activation	Relu
Solver	Adam
Alpha	0.0001
Batch_size	Auto
Learning_rate	Constant
Learning_rate_init	0.001
Max_iter	50000
Shuffle	True
Random_state	0
Tol	0.0001
Warm_start	False
Early_stopping	False
Beta_1	0.9
Beta_2	0.999
Epsilon	1e-08
N_iter_no_change	10

Table S2. Patient and lesion characteristics according to the treatment.

	Surgery	ESD with additional surgery	ESD alone	*P* value
	n = 2970	n = 815	n = 257	
Age, years	70 (26 - 94)	71 (39 - 92)	78 (37 - 92)	< 0.0001
Sex				< 0.0001
Male	1863 (63)	627 (77)	184 (72)	
Female	1107 (37)	188 (23)	73 (28)	
Location				< 0.0001
Upper	450 (15)	195 (24)	67 (26)	
Middle	1465 (49)	340 (42)	110 (43)	
Lower	1055 (36)	280 (34)	80 (31)	
Size, mm	33 (3 - 185)	23 (4 - 110)	24 (2 - 115)	< 0.0001
Invasion depth				< 0.0001
M	760 (26)	122 (15)	79 (31)	
SM1	331 (11)	115 (14)	52 (20)	
SM2	1879 (63)	578 (71)	126 (49)	
Histologic type				
Differentiated type				0.07
tub1 dominant	674 (22)	348 (42)	134 (52)	
tub2 dominant	408 (13)	179 (22)	59 (23)	
pap dominant	51 (2)	21 (3)	3 (1)	

Undifferentiated type				0.01
por dominant	741 (25)	51 (6)	15 (6)	
sig dominant	357 (12)	47 (6)	14 (6)	
muc dominant	10 (1)	1 (1)	0 (0)	
Mixed type				0.004
Differentiated type dominant	440 (15)	122 (15)	24 (9)	
Undifferentiated type dominant	289 (10)	46 (5)	8 (3)	
Lymphatic involvement				< 0.0001
Positive	998 (34)	313 (38)	55 (21)	
Negative	1972 (66)	502 (62)	202 (79)	
Vascular involvement				< 0.0001
Positive	587 (20)	129 (16)	24 (9)	
Negative	2383 (80)	686 (84)	233 (91)	
Ulcerlation [a, b]				0.22 [c]
Positive	-	207 (27)	71 (31)	
Negative	-	560 (73)	157 (69)	
Vertical margin [a]				0.06 [c]
Positive	-	109 (13)	27 (10)	
Negative	-	663 (81)	207 (81)	
Unclear	-	43 (6)	23 (9)	

Lymph node metastasis				< 0.0001
Positive	463 (16)	67 (8)	12 (5)	
Negative	2507 (84)	748 (92)	245 (95)	

Data are expressed as the median (range) or number (%).

[a] Shows only the results of the lesions resected by ESD with/without additional surgery.

[b] Data were not available for 48 and 29 lesions in ESD with additional surgery and ESD alone groups, respectively.

[c] Statistical tests were performed between ESD with additional surgery group and ESD alone group.

ESD, endoscopic submucosal dissection; M, limited to mucosa; SM1, submucosal invasion < 500 μm; SM2, submucosal invasion ≥ 500 μm; tub1, well differentiated tubular adenocarcinoma; tub2, moderately differentiated tubular adenocarcinoma; pap, papillary adenocarcinoma; por, poorly differentiated adenocarcinoma; sig, signet-ring cell carcinoma; muc, mucinous adenocarcinoma.

Table S3. Characteristics of true negatives at cut-off scores where machine learning model identified patients with lymph node metastasis with 100% sensitivity (n = 120).

Age, years	70 (43 - 89)
Sex	
Male	89 (74)
Female	31 (26)
Location	
Upper	39 (33)
Middle	60 (50)
Lower	21 (17)
Size, mm	23 (5 - 72)
Invasion depth	
M	83 (69)
SM1	6 (5)
SM2	31 (26)
Histologic type	
Differentiated type	
tub1 dominant	38 (32)
tub2 dominant	39 (33)
pap dominant	2 (2)
Undifferentiated type	
por dominant	10 (8)
sig dominant	25 (21)
muc dominant	0 (0)
Mixed type	
Differentiated type dominant	1 (1)
Undifferentiated type dominant	5 (3)
Lymphatic involvement	
Positive	0 (0)
Negative	120 (100)
Vascular involvement	
Positive	4 (3)
Negative	116 (97)
Ulceration [a]	
Positive	52 (43)
Negative	64 (57)

Vertical margin	
Positive	14 (12)
Negative	100 (83)
Unclear	6 (5)
eCura system score	
0	49 (41)
1	68 (57)
2	3 (2)
3 or more	0 (0)
Lymph node metastasis	
Positive	0 (0)
Negative	120 (100)

Data are expressed as the median (range) or number (%).

[a] Data were not available for 4 lesions.

ESD, endoscopic submucosal dissection; M, limited to the mucosa; SM1, submucosal invasion < 500 μm; SM2, submucosal invasion≥ 500μm; tub1, well-differentiated tubular adenocarcinoma; tub2, moderately differentiated tubular adenocarcinoma; pap, papillary adenocarcinoma; por, poorly differentiated adenocarcinoma; sig, signet-ring cell carcinoma; muc, mucinous adenocarcinoma

Figure1

Click here to access/download;Figure;Figure 1_R1.tif

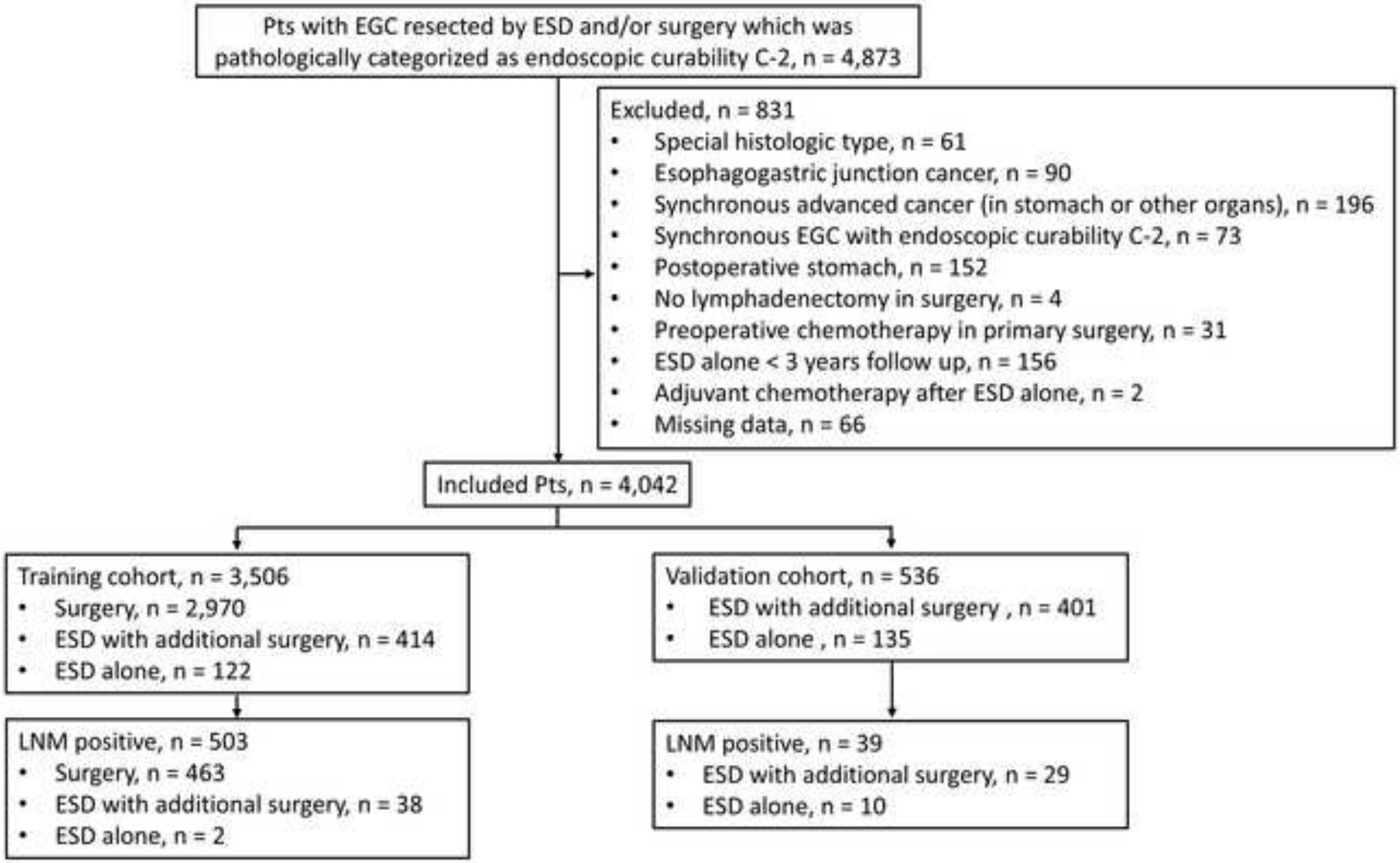

Figure2

Click here to access/download;Figure;Figure 2.TIF

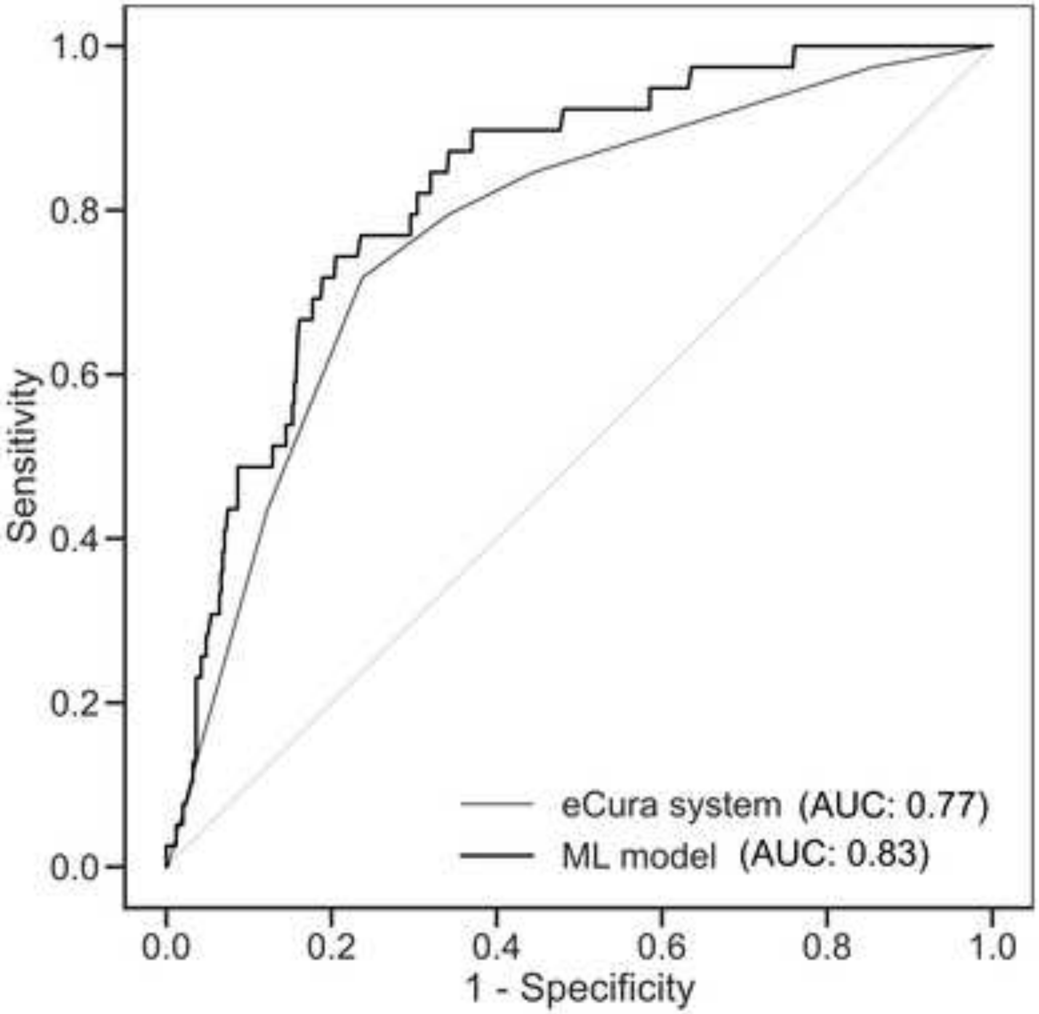

Figure3 Click here to access/download;Figure;Figure 3.TIF

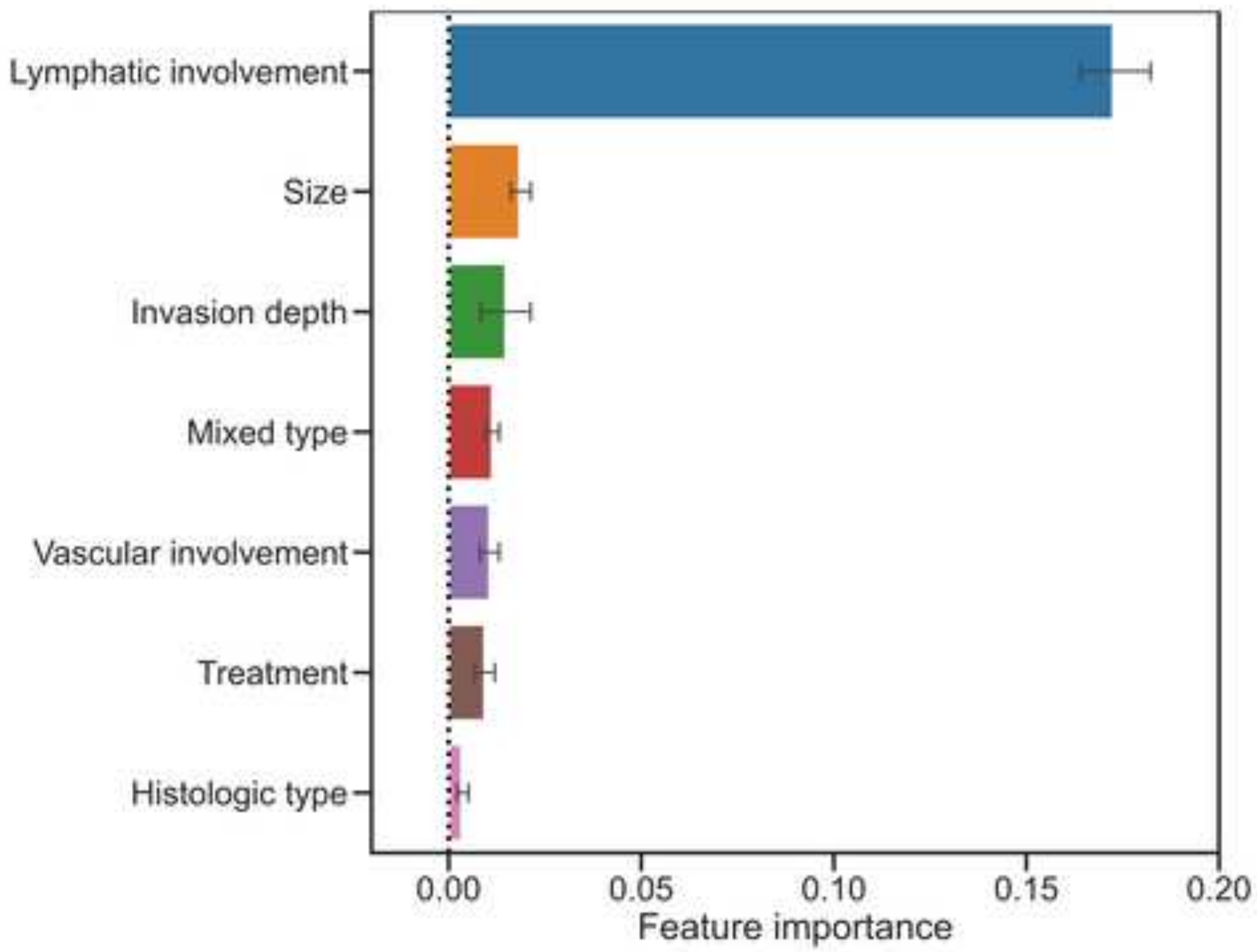

Mini-abstract: We developed a neural network-based machine learning model that predicts the risk of lymph node metastasis in patients with early gastric cancer who did not meet the endoscopic curability criteria.

ICMJE DISCLOSURE FORM

**Date:**	12/6/2023
**Your Name:**	Tetsuo Takehara
**Manuscript Title:**	A machine learning model for predicting the lymph node metastasis of early gastric cancer not meeting the endoscopic curability criteria
**Manuscript Number (if known):**	Click or tap here to enter text.

In the interest of transparency, we ask you to disclose all relationships/activities/interests listed below that are related to the content of your manuscript. "Related" means any relation with for-profit or not-for-profit third parties whose interests may be affected by the content of the manuscript. Disclosure represents a commitment to transparency and does not necessarily indicate a bias. If you are in doubt about whether to list a relationship/activity/interest, it is preferable that you do so.

The author's relationships/activities/interests should be defined broadly. For example, if your manuscript pertains to the epidemiology of hypertension, you should declare all relationships with manufacturers of antihypertensive medication, even if that medication is not mentioned in the manuscript.

In item #1 below, report all support for the work reported in this manuscript without time limit. For all other items, the time frame for disclosure is the past 36 months.

		**Name all entities with whom you have this relationship or indicate none (add rows as needed)**	**Specifications/Comments (e.g., if payments were made to you or to your institution)**
		Time frame: Since the initial planning of the work	
1	All support for the present manuscript (e.g., funding, provision of study materials, medical writing, article processing charges, etc.) **No time limit for this item.**	☒ None	
			Click the tab key to add additional rows.
		Time frame: past 36 months	
**2**	Grants or contracts from any entity (if not indicated in item #1 above).	☒ **None**	
**3**	Royalties or licenses	☒ **None**	

		Name all entities with whom you have this relationship or indicate none (add rows as needed)	Specifications/Comments (e.g., if payments were made to you or to your institution)
**4**	Consulting fees	☒ **None**	
**5**	Payment or honoraria for lectures, presentations, speakers bureaus, manuscript writing or educational events	☒ **None**	
**6**	Payment for expert testimony	☒ **None**	
**7**	Support for attending meetings and/or travel	☒ **None**	
**8**	Patents planned, issued or pending	☒ **None**	
**9**	Participation on a Data Safety Monitoring Board or Advisory Board	☒ **None**	
**10**	Leadership or fiduciary role in other board, society, committee or advocacy group, paid or unpaid	☒ **None**	

		Name all entities with whom you have this relationship or indicate none (add rows as needed)	Specifications/Comments (e.g., if payments were made to you or to your institution)
**11**	Stock or stock options	☒ **None**	
**12**	Receipt of equipment, materials, drugs, medical writing, gifts or other services	☒ **None**	
**13**	Other financial or non-financial interests	☒ **None**	

Please place an "X" next to the following statement to indicate your agreement:

☒ I certify that I have answered every question and have not altered the wording of any of the questions on this form.
